# Uterine Didelphys with Transverse Vaginal Septum – A Complex Rare Müllerian Anomaly

**DOI:** 10.15388/Amed.2021.28.2.2

**Published:** 2021-07-29

**Authors:** Dina Aisha Khan, Nalini Sharma, Anusmita Saha, Rituparna Das, Subrat Panda

**Affiliations:** Department of Obstetrics and Gynaecology, Hamdard Institute of medical sciences and research, New Delhi, India; Department of Obstetrics and Gynaecology, North Eastern Indira Gandhi Regional Institute of Health and Medical Sciences, Shillong, Meghalaya, India https://orcid.org/ID-0000-0001-5462-3017; Department of Obstetrics and Gynaecology, North Eastern Indira Gandhi Regional Institute of Health and Medical Sciences, Shillong, Meghalaya, India; Department of Obstetrics and Gynaecology, North Eastern Indira Gandhi Regional Institute of Health and Medical Sciences, Shillong, Meghalaya, India; Department of Obstetrics and Gynaecology, North Eastern Indira Gandhi Regional Institute of Health and Medical Sciences, Shillong, Meghalaya, India

**Keywords:** Müllerian duct anomaly, uterine didelphys, transverse vaginal septum, primary amenorrhoea, dysmenorrhoea

## Abstract

During the development of the female genital tract, any insult to the normal development process results in a set of intriguing abnormalities known as Müllerian duct abnormalities. The uterine didelphys is the second least common type of anomaly among these, which may commonly be associated with a longitudinal vaginal septum (lateral fusion defect). However uterine didelphys along with a transverse vaginal septum (lateral fusion plus resorption defect) is a very rare finding and to the best of our knowledge, thecase that we hereby report is the second one in literature.

A 16-year-old unmarried girl presented with primary amenorrhoea and cyclical pain for 18months.On clinical examination and imaging, a case of uterine didelphys and transverse vaginal septum was found. Her urinary tract was normalon USG and MRI evaluation. Excision of the septum was done by abdomino-vaginal approach. The patient was discharged well.

We conclude that a patient presenting with primary amenorrhea especially with cyclical dysmenorrhea with a transverse vaginal septum on examination should be thoroughly investigated for associated upper genital tract abnormalities as the treatment strategy and prognosis is largely dependent on the correct classification of the anomaly.

## Abstract (lt)

**Dviguba gimda su skersine makšties pertvara. Sudėtinga retesnė Miulerio latakų anomalija:**

**Santrauka. **Sutrikus moters lyties organų įprastam vystymuisi, atsiranda intriguojančių anomalijų, vadinamų Miulerio latakų anomalijomis. Dviguba gimda yra antra pagal retumą tarp šių anomalijų ir jai dažniausiai būdinga išilginė makšties pertvara (šoninio sujungimo defektas). Mūsų žiniomis, dviguba gimda su skersine makšties pertvara (šoninio sujungimo ir įsiurbimo defektu) pasitaiko labai retai – tai tik antras atvejis, aprašytas literatūroje.

Šešiolikos metų netekėjusi mergina kreipėsi dėl pirminės amenorėjos ir menstruacinių skausmų, trunkančių 18 mėnesių. Atlikus klinikinę apžiūrą ir panaudojus medicininę atvaizdavimo techniką, nustatyta dviguba gimda ir skersinė makšties pertvara. Ištyrus ultragarsu ir atlikus MRT, anomalijų šlapimtakiuose nenustatyta. Pertvara pašalinta atlikus pilvo ir makšties operaciją. Išrašoma pacientė jautėsi gerai.

Darome išvadą, kad pacientė, kuriai buvo pirminė amenorėja, ypač jei yra dismenorėja ir apžiūros metu nustatoma skersinė makšties pertvara, turėtų būti nuodugniai ištirta dėl galimų viršutinių lyties organų anomalijų, nes teisingai nustačius anomaliją parenkamas tinkamas gydymas ir nustatoma teisinga prognozė.

**Raktažodžiai:** Miulerio latakų anomalija, dviguba gimda, skersinė makšties pertvara, pirminė amenorėja, dismenorėja

## Introduction

Abnormalities of the female genital tract may be simple or complex and occur from defects at any of the steps of thedevelopment of Müllerian ducts. The true prevalence of malformation remains uncertain as many of them are asymptomatic and are not picked up. Uterine malformation has been reported in up to 7% of the general population, 7% of the infertile population, and 18% of those withrecurrent pregnancy loss [1].

Grimbizis et al. observed the septate uterus as the most commonanomaly accounting for 35% of the Müllerianduct anomalies.Other anomalies include bicornuateuterus – 25%, arcuate – 20%, unicornuate – 9.6%, and complete agenesis – 3%. Didelphys uterus is the second least commonconstituting 8.3% of all Müllerian duct anomalies (MDA) [2]. These anomalies resultfrom the failure of development, fusion, canalization, or reabsorption of the Müllerianducts at 6 to 22weeks of intrauterine life.

Uterus didelphys (double uterus) results fromcomplete failureof the Müllerianducts to fuse leading to two separateuterine cavities and two cervices. It has a reported prevalenceof 0.03% to 0.1% and accounts for 5% of all Mülleriananomalies [3]. Two cervicesmust be recognized eithersonographically or by clinical examination to confirmuterus didelphys.Similarly, MRI demonstrates two separate uterine bodieswith divergent apices and two separate cervices [4]. Comparableto other Müllerian anomalies, MRI hasa high degree of sensitivity (100%) and specificity(79%), as well as a high degree of agreement (100%)when compared with hysteroscopy, laparoscopy, and laparotomy[5].

A transverse vaginal septum is a rare abnormality of the female genital tract with an incidence between 1/2100 and 1/7200 [6]. The most common etiology is a defect in the fusion and/or channeling of the vaginal plate or failure of the paramesonephric ducts to congregate the urogenital sinus [7].

The most common location of the transverse vaginal septumis in the lower part of the vagina. 72% of the septums are in the lower part of the vagina, 22% in the central part, and only 6% are in the upper part [8].Vaginal anomalies are diagnosed mostly during the gynecological examination and MRI is used in cases with an obstructive anomaly to find out any associated anomaly.

The association of renal abnormalities with Müllerian anomalies is well recognized and consequently, the assessment of the renal tract forms part of the routine assessment of patients presenting with Müllerian anomalies. In the largest series reporting the incidence of renal anomalies in patients with MDAs, renal agenesis has been found in around 30% of patients [9].

Other abnormalities of the renal tract found in these patients included pelvic kidneys, malrotation, dysplastic kidneys, multicystic dysplastic kidney,ectopic ureter, etc.

It is not uncommon to find a longitudinal vaginal septum along with uterine didelphys.

However, we here present a rare case of the transverse vaginal septum with uterine didelphys. In literature, only one case has been reported to date. To the best of our knowledge, this is the second case.

A patient with a transverse vaginal septum and uterine didelphys presents with primary amenorrhea and cyclical pain or pain in the lower abdomen. Diagnosis is clinically aided with imaging modalities like ultrasonography. However magnetic resonance imaging (MRI) is the investigation of choice because of its high precision and elaborated delineation of uterovaginal anatomy. Laparoscopy is mainly needed wherein interventional therapy is likely to be undertaken.

## Case Report

**Figure 1.**
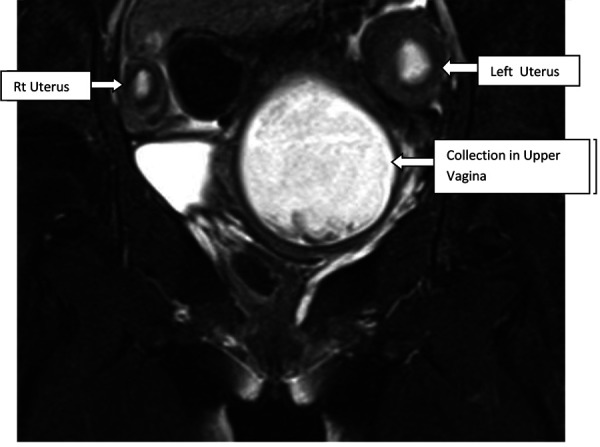
T2 coronal showing twouteruses. Upper vaginal cavity is distended with hyperintense collection

**Figure 2.**
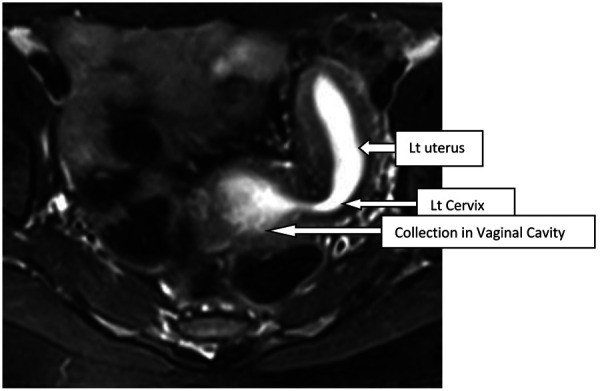
T2 axial shows dilated endometrial cavity of uterus communicating with collection of upper vaginal cavity

A 16-year-old unmarried girl presented to theoutpatient department of Obstetrics and Gynaecology, NEIGRIHMs Shillong with primary amenorrhoea and cyclical abdominalpain for 18months. The pain was moderate to severe in intensity, spasmodic in nature, and relieved by NSAIDs. She was a known case of juvenile diabetes mellitus. On general physical examination, the patient was thin built with the development of breast along with axillary and pubic hair, classified as Tanner’s stage 4. Her vitals were normal with normal findings on respiratory, cardiovascular, central nervous system examination. Her abdomen was soft and nontender with no mass palpable. After counseling and obtaining consent from the patient and her guardians, per vaginal examination was performed using one finger, which revealed an obstruction in the proximal part of the vagina(tranverse vaginal septum). A bulge was felt towards the left side of the vagina. Perrectal examination revealed a bulge in the upper part. She was subjected to radiological evaluation to look for other anomalies in the uppergenitourinary system.Transabdominal ultrasonography showed uterine didelphys with hematometra involving the right horn and normal renal system. She further underwent an MRI pelvis, which revealed two widely separated uterus with separate cervices. Both uteri were mildly distended with blood products, lesser in the left uterus with two cervices seen. There was evidence of a vaginal septum of 4mm thickness with a distended upper vaginal cavity and collapsed lower vagina. (Figure 1, 2) Her urinary system was normal.

**Figure 3.**
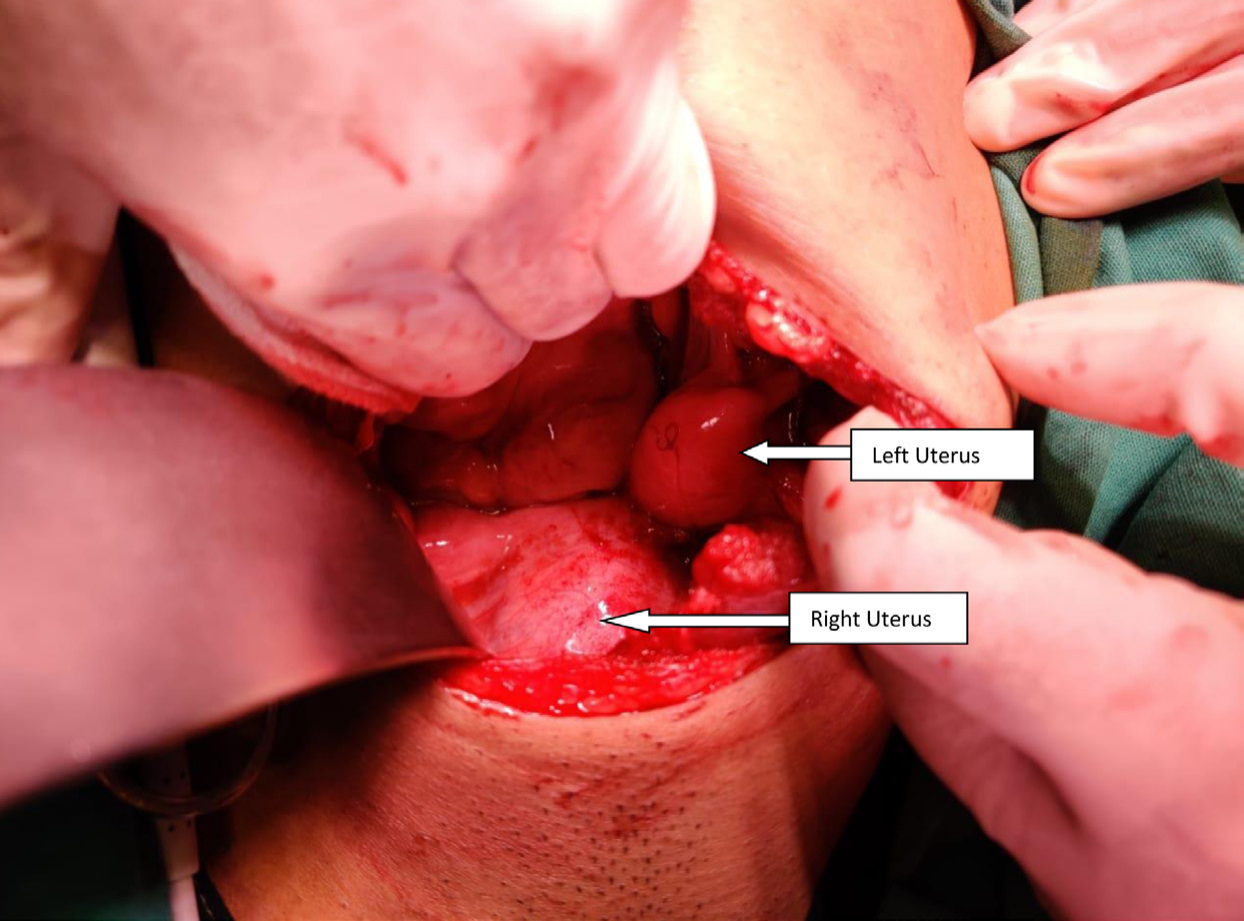
Centrally placed – distended right uterus with normal size(bulky) left uterus

**Figure 4.**
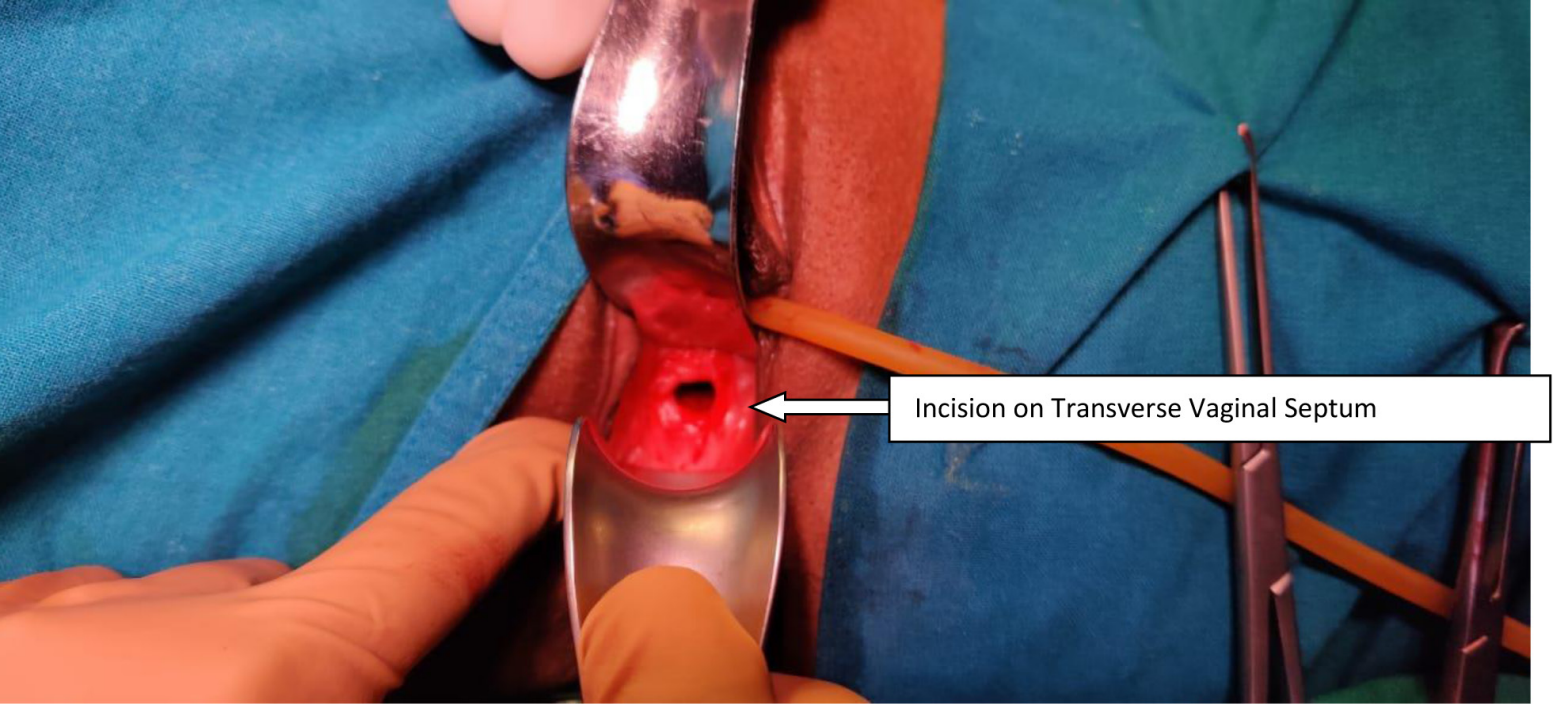
After excision of transverse vaginal septum

The patient was planned and posted for excision of transverse vaginal septum. Initially, ultrasonography-guided pervaginal excision of the transverse vaginal septum was tried after aspiration with a needle. Even after extensive dissection continuity between the cervices and vagina could not be establishedand a decision for the abdominal approach was taken (preoperative consent was obtained). On laparotomy, a midline distended uterine horn was seen which was identified as the right uterus(Figure 3), with a distended fallopian tube on the same side. Left-sided uterus(slightly bulky in size), left fallopian tube and both ovaries were normal. Flimsy adhesions and altered colored blood were noted in the pelvis due to retrograde bleeding. A midline incision was given on the right uterus after pushing the bladder down and dark altered menstrual blood evacuated. A dilator was guided into the incision. The further incision was given vaginally on the vaginal septum over the dilator and communication was established between theproximal and middle vagina(Figure 4).A Foley catheter with a distended bulb was kept in the endometrial cavity for maintaining patency of the track. Another uterus was located on the left side, no continuity with the newly created opening was seen. The patient’s parents didn’t want further intervention.Counseling was done regarding the possibility of recollection and the need for further intervention.The immediate postoperative period was uneventful. 

On postoperative day 14, the Foley catheter kept in the uterine cavity was removed and a vaginal mold was inserted. The patient’s blood sugar level during the postoperative period was managed accordingly. She recovered well and was discharged subsequently. She had one episode of menstruation afterward.

## Discussion

Obstructive structural anomaliesof the reproductive tractcan cause hematocolpos, hematometrawhich can present asprimary amenorrhoea with cyclic pelvic pain shortly after the age of menarche in adolescent girls.The case discussed here had a typical presentation and an obstruction was diagnosed at the level of the proximal vagina with the presence of hematocolpos indicating intact cervix along with the concurrence of another uterine anomaly, uterine didelphys. 

The embryological development of female urogenital organs is a complex and closely related process. The female genital organs are derived from the paramesonephric duct also known as the Müllerian duct. At the 8th week of intrauterine life, the Müllerian duct appears (invagination of the coelomic epithelium) and develops close to the mesonephric (Wolffian) ducts and grows toward the urogenital sinus, forming the 2 uterovaginal canals. At the 11th week, these ducts fuse (lateral fusion), to form a single canal, eventually giving rise to bilateral fallopian tubes, uterus, cervix, and upper part of the vagina. In the interim, the sinovaginal bulbs invaginate from the urogenital sinus which moves cranially to fuse with sinus tubercle on the caudal end of the Müllerian ducts and forms the vaginal plate (vertical fusion). This plate undergoes resorption to form a canalized vagina; the process of resorption is completed by 24 weeks of gestation. When any of the above-mentioned processes is disrupted, abnormalities of genital structures occur. Considering the embryological development the case discussed mandates two different processes to be disrupted. Uterine didelphys is the result of a defect in lateral fusion while the transverse vaginal septum is a result of a defect in resorption. Such an anomaly coexisting in a single patient is not only very rare but also needs to be addressed separately while managing the patient. 

The fertility of women is a problem in such cases, firstly due to amenorrhoea, dyspareunia,and associated endometriosis caused by the septum. Secondly, due to uterine didelphys the risks of miscarriage, fetal growth restriction and prematurity are increased. The reproductive potential of uterine didelphys is worse than that of a normal uterus but better than other uterine anomalies [10]. 

Uterine anomalies have been classified according to the American Fertility Society (AFS) which divides uterine malformations into seven main groups. This system does not include vaginal anomalies and certain combined anomalies[11].As per the AFS classification the present case falls inclassIII.

By the ESHRE (European) classification, all anomalies of the female genital tract can be grouped. One big advantage in the new classification is that it also covers malformations of the uterine cervix and the vagina [12].According to the European classification the present casefalls in the category of U3C2V3.

Treatment modalities are case-specific. Transverse vaginal septum excision is to be undertaken for treating primary amenorrhea, pain from hematometrocolpos, and dyspareunia. In this case, the plan was to excise the septum by vaginal route but on extensive dissection connectivity between cervix and septum could not beestablished and thus the decision was taken to adopt an abdominal vaginal approachas suggested by van Bijsterveldt [13].

Postoperative vaginal stenosis remains the most common complication. Postoperative vaginal dilation may help to reduce scarring and stenosis at the surgical site.

Surgical correction of didelphys uterus (metroplasty) is usually not indicated as uterus didelphys have a good reproductive prognosis.However, a retrospective study done by Zhang et al. demonstrated that patients with didelphys uterus required infertility treatments more frequently than with other anomalies [14]. Intervention is required in cases of OHVIRAsyndrome since one of the vaginae is obliterated [15].

Moawad et al. reported a case of a 15-year-old girl with abdominal mass with primary amenorrhoea and found a combined uterus didelphys, double cervix with a longitudinal and transverse vaginal septum [16].

Eric reported a case of the complete septate uterus with a longitudinal and transverse vaginal septum [17] while B. M. Carrington et al. in 1990 mentioned in their article about a patient with uterine didelphys having obstructive transverse vaginal septum in the upper one-third of the vagina [18]. That is the only case of uterine didelphys with transverse vaginal septum found in a literature search. 

Our case is unique as cases reported mainly had longitudinal vaginal septum with uterus didelphys and rarely both longitudinal and transverse vaginal septum. A combination of uterine didelphys with only transverse vaginal septum is the rarest. To the best of our knowledge, this is the second reported case.

## Conclusion

Didelphys uterus is a very rare uterine anomaly, can be associated with a longitudinal vaginal septum. Combination with transverse vaginal septum makes it even rarer. Management in such cases is challenging as nothing has been established.The treatment strategy is to provide symptomatic relief with the relief of obstruction aimingfor the best reproductive outcome. Individualization is the crux of management.By reporting this case we hope to alert gynecologists regarding the rarest possibility. 
